# Dietary Fructose Alters Duodenal Mucin Glycosylation and Mucus Production in High-Fat Diet-Fed Mice

**DOI:** 10.3390/ijms27104189

**Published:** 2026-05-08

**Authors:** Donatella Mentino, Alessia Provera, Cristina Vecchio, Alessandro Antonioli, Anteneh Nigussie Sheferaw, Nastasia Taldone, Rossella Vitale, Chiara Passamonti, Stefania Fensore, Flavia Prodam, Salvatore Sutti, Maria Mastrodonato, Maria Felicia Faienza

**Affiliations:** 1 Department of Biosciences, Biotechnology and Environment, University of Bari Aldo Moro, 70125 Bari, Italy; n.taldone@alumni.uniba.it (N.T.); maria.mastrodonato@uniba.it (M.M.); 2 Department of Health Sciences, University of Piemonte Orientale, 28100 Novara, Italy; 3 Pediatric Unit, Department of Precision and Regenerative Medicine and Ionian Area, University of Bari Aldo Moro, Piazza G. Cesare 11, 70124 Bari, Italymariafelicia.faienza@uniba.it (M.F.F.); 4 Department of Mathematics, University of Luxembourg, 4365 Luxembourg, Luxembourg; 5 Department of Socio-Economic, Managerial and Statistical Studies, University of Chieti-Pescara, 65129 Pescara, Italy; 6 Water Research Institute (IRSA), National Research Council of Italy (CNR), 74123 Taranto, Italy

**Keywords:** fructose, high-fat diet, lectins, intestinal glycosylation patterns, age-related intestinal changes

## Abstract

Fructose, a key component of modern diets, is closely linked to the growing prevalence of pediatric obesity and metabolic alterations. Although numerous studies highlight its systemic consequences, including altered carbohydrate and lipid metabolism and increased cardiovascular risk, the direct impact of fructose, particularly its role in modulating mucin composition, a key determinant of the mucosal barrier, remains poorly explored. This study investigated whether fructose supplementation modifies high-fat diet (HFD)-induced changes in duodenal mucin production and whether these effects vary depending on age in animals. To this end, young and adult mice were fed a normal diet (ND), HFD, or an HFD supplemented with 30% fructose (*w*/*v*) in drinking water (HFD+Fru) for 16 weeks. Brunner’s glands and villus goblet cells were then analyzed using conventional histochemistry and a panel of lectins to evaluate possible alterations in intestinal mucus glycosylation. Results showed that both HFD and HFD+Fru significantly increased body weight. In young mice, HFD+Fru induced a compensatory mucosal phenotype characterized by increased villus PAS (Periodic Acid–Schiff) reactivity (2% vs. ND), elevated sialylated mucin secretion rate (SSR) in Brunner’s glands (25% vs. ND) and villi (17% vs. ND), and higher SNA (up to 46% vs. ND) and PNA (up to 39% vs. ND) in villus goblet cells. In contrast, adult mice receiving HFD+Fru exhibited a maladaptive response, characterized by a reduction in villus PAS-positive mucins (6% vs. ND), decreased villus SSR (5% vs. ND), diminished sialylation (up to 43% SNA vs. ND) and GlcNAc (up to 50% reduction in WGA vs. ND) in villus goblet cells, and marked loss of fucosylation in Brunner’s glands (81% vs. ND) and villus goblet cells (66% vs. ND). These results reveal that fructose-enriched HFD remodels duodenal mucin O-glycosylation in an age-dependent manner, suggesting that while young mice exhibit transient adaptive responses, prolonged exposure can deplete these mechanisms, leading to a compromised adult epithelial barrier. This age-specific vulnerability may significantly contribute to the pathogenesis of diet-related intestinal disorders and obesity-related complications in later life, highlighting the need for early dietary interventions.

## 1. Introduction

Fructose, the sweetest natural sugar, now floods soft drinks, juices, and baked goods, key components of modern diets, especially for children and adolescents. Its consumption has surged over the past 40 years, paralleling the rise in pediatric obesity [1,2]. High consumption of simple sugars, particularly fructose from added sugars and high-fructose corn syrup (HFCS), is considered a key dietary factor in the development of metabolic syndrome and intestinal disorders [3]. Unlike glucose, fructose is predominantly metabolized in the liver, where high intake promotes de novo lipogenesis, triglyceride accumulation, insulin resistance, hyperuricemia, and dyslipidemia, leading to systemic energy homeostasis disruption [1,4]. Numerous experimental and clinical studies have investigated the systemic consequences of high-fructose diets, highlighting modifications in glucose and lipid metabolism, low-grade chronic inflammation, and increased cardiovascular risk. However, direct effects of fructose on the intestinal mucosa remain underexplored, despite the intestine being the primary site of exposure for dietary nutrients. It remains poorly understood how fructose supplementation in high-fat diet (HFD) mice affects the composition and distribution of mucins [1,5,6]. In this context, Brunner’s glands should not be considered merely mucus-secreting structures but rather dynamic components of the duodenal environment that actively respond to dietary stimuli. Emerging evidence suggests that excess fat and sugar intake can alter mucus composition, making these glands valuable bioindicators of early tissue alterations induced by unbalanced diets, as observed in goblet cells of the duodenal villi. Due to their strategic location at the interface between luminal contents and the mucus layer, Brunner’s glands and goblet cells represent an ideal model for investigating diet-induced changes in mucin glycosylation [7,8]. Modifications in mucin glycosylation patterns and secretion dynamics have been associated with increased intestinal permeability observed in HFD models [7,9]. Previous studies demonstrate that HFD modifies duodenal mucin glycosylation, characterized by increased Brunner’s gland secretory activity and reduced fucosylated/sulfated residues in both glandular and villus goblet cell mucins. Whether fructose supplementation further modifies these patterns and whether such effects occur in an age-dependent manner remain to be investigated.

Aging is associated with progressive alterations in intestinal homeostasis, including reduced epithelial renewal, impaired stem cell function, increased susceptibility to endoplasmic reticulum (ER) stress, and changes in mucus barrier integrity. These processes are accompanied by alterations in goblet cell function, mucin biosynthesis, and host–microbiota interactions, which together may critically influence the intestinal response to dietary challenges. In this context, younger individuals are generally characterized by greater epithelial plasticity and adaptive capacity, whereas more mature stages show reduced resilience to metabolic and proteotoxic stress [10,11]. Based on these considerations, we hypothesized that the impact of a fructose-enriched high-fat diet on mucin production and glycosylation would differ between developmental stages, reflecting distinct adaptive versus maladaptive responses.

Currently, no data are available regarding modifications in mucin composition and distribution within duodenal villi and Brunner’s glands in murine models subjected to a normal diet (ND), an HFD, or a high-fat diet supplemented with fructose (HFD+Fru) [7,12]. To investigate developmentally distinct stages relevant to human growth, we compared early post-weaning (young) mice with late-adolescent mice (postnatal day 42) [13]. This study aimed to evaluate whether fructose supplementation in an HFD exacerbates deleterious metabolic effects and induces structural alterations in the duodenum, comparing young and adult mice. This approach aims to elucidate age-dependent mechanisms through which fructose promotes systemic metabolic dysfunction characteristic of Western dietary patterns [14].

## 2. Results

**Figure 1:**
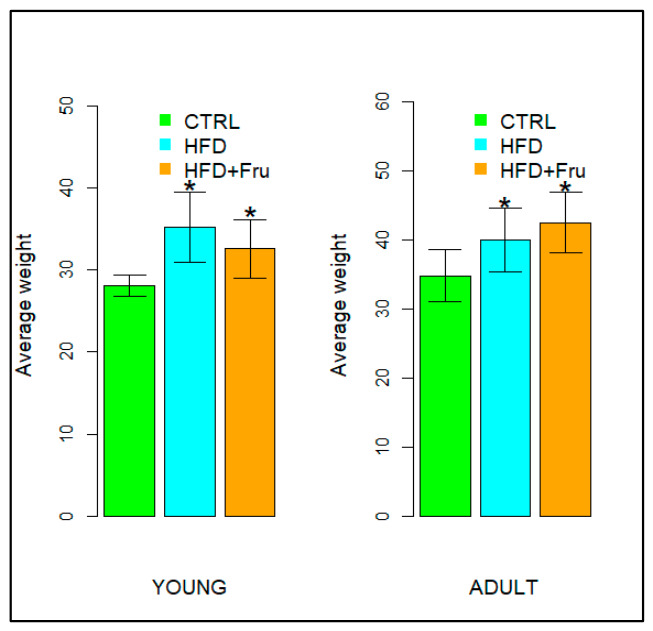
Body weight of young and adult mice fed a normal diet (ND), a high-fat diet (HFD), or a high-fat diet supplemented with fructose (HFD+Fru). Data are presented as means ± SD. Statistical analyses were performed using one-way Welch’s ANOVA for young mice and one-way ANOVA for adult mice. An asterisk (*) indicates a statistically significant difference from the ND group (*p*-value < 0.05).

Exposure to high-fat and fructose-enriched diets was associated with a significant increase in body weight in both young and adult mice. Specifically, in young mice, final body weight was significantly higher in the HFD group (35.21 ± 4.25 g) compared with the ND group (28.09 ± 1.33 g), corresponding to an approximately 25.3% increase. The HFD+Fru group also exhibited a higher final body weight (32.59 ± 3.60 g), representing a 16.0% increase relative to ND and indicating an intermediate effect between the two dietary regimens. In adult mice, body weight was significantly higher both in the HFD (39.93 ± 4.58 g) and HFD+Fru (42.45 ± 4.35 g) groups compared with the ND group (34.72 ± 3.77 g), with an increase of 15% and 22.3%, respectively (Figure 1) (Table 1).

### 2.1. Conventional Histochemistry

#### 2.1.1. PAS to Characterize Acidic and Neutral Glycoconjugates

**Figure 2:**
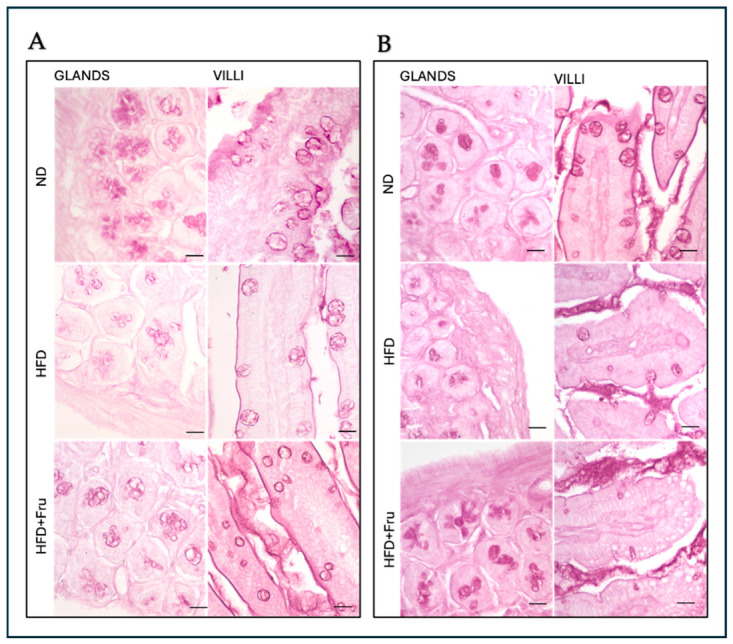
(**A**) PAS method on young mice to detect the presence of glycoproteins in Brunner’s glands and in villus goblet cells. (**B**) PAS method on adult mice to detect the presence of glycoproteins in Brunner’s glands and in villus goblet cells. Scale bar: 20 µm.

Histological analysis of intestinal sections stained with H&E (hematoxylin–eosin) revealed prominent lymphoid nodules in the intestinal wall of mice fed HFD and HFD+Fru, which were absent in ND-fed mice. In addition, Clear age-related differences in duodenal villus architecture were observed. In young mice, villi appeared long and slender, forming tall finger-like projections into the lumen, whereas in adult mice, villi were shorter, broader, and frequently showed vacuolated enterocytes, giving them a blunted, stocky appearance. Complementary staining with PAS demonstrated an abundant glycoprotein content in the intestinal mucosa of both young and adult mice under all dietary regimens (ND, HFD, and HFD+Fru), consistent with a widespread expression of neutral mucins and other PAS-reactive glycoconjugates throughout the epithelium and associated glands (Figure 2A,B).

**Figure 3:**
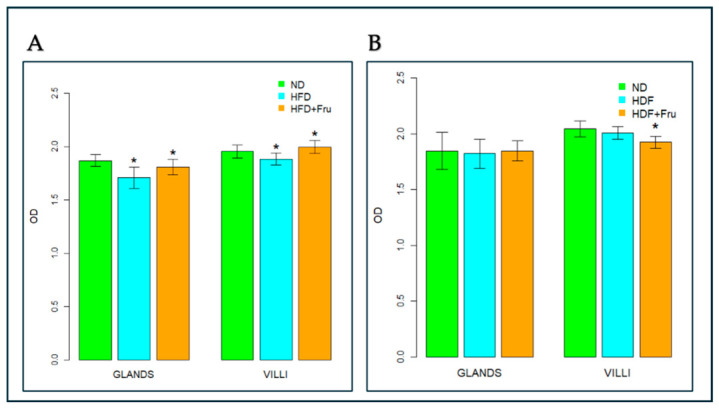
Analysis of PAS staining in Brunner’s glands and villus goblet cells of young and adult mice. (**A**) Young mice and (**B**) adult mice fed a normal diet (ND), high-fat diet (HFD), or high-fat diet supplemented with fructose (HFD+Fru). PAS staining was quantified to evaluate glycoprotein content in Brunner’s glands and villus goblet cells. Data are presented as means ± SD. Statistical analyses were performed using ANOVA for young mice (**A**) and the Kruskal–Wallis test for adult ones (**B**). An asterisk (*) indicates a statistically significant difference from the ND group (*p*-value < 0.05).

In young mice, Brunner’s glands showed a statistically significant reduction in PAS intensity in both the HFD and HFD+Fru groups compared with ND, by 9% and 3%, respectively, indicating a lower amount of stored glycoproteins in the glands (Figure 3A). Villus goblet cells showed a significant 4% decrease in PAS staining intensity in the HFD group compared with ND, whereas the HFD+Fru group exhibited a significant 2% increase relative to ND (Figure 3A).

In adult mice, Brunner’s glands showed no appreciable change in PAS positivity across the experimental diets, suggesting that glycoprotein content in these glands remained unchanged (Figure 3B). Villus goblet cells exhibited reduced PAS staining intensity in the HFD+Fru group compared with ND, indicating a lower glycoprotein content of approximately 6% in these cells (Figure 3B).

#### 2.1.2. Alcian Blue pH 2.5 and High Iron Diamine to Characterize Sialylated and Sulfated Glycoconjugates

**Figure 4:**
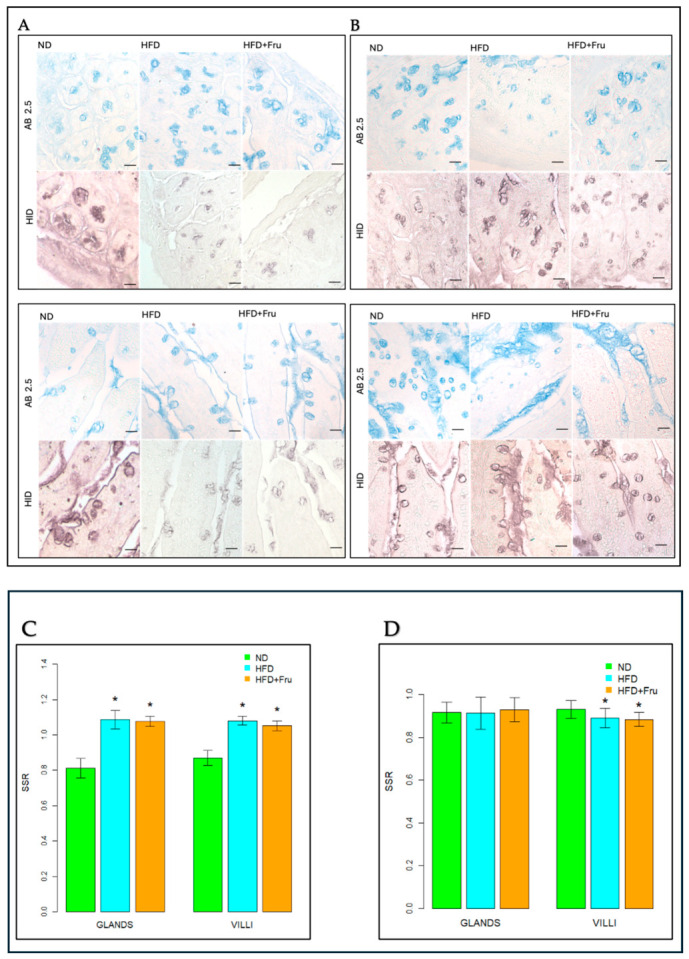
(**A**) Alcian blue at pH 2.5 and high iron diamine in young mice to detect sialylated and sulfated acidic glycoproteins in Brunner’s glands and in villus goblet cells. Scale bar: 20 µm. (**B**) Alcian blue at pH 2.5 and high iron diamine in adult mice to detect sialylated and sulfated acidic glycoproteins in Brunner’s glands and in villi goblet cells. Scale bar: 20 µm. (**C**) Ratio between AB pH 2.5/HID in young mice, shown as means ± SD, analyzed by one-way ANOVA for glands and Kruskal–Wallis for villi. (**D**) Ratio between AB pH 2.5/HID in adult mice, shown as means ± SD, analyzed by ANOVA. An asterisk (*) indicates a statistically significant difference in mean value compared with the ND group (*p*-value < 0.05).

Alcian blue pH 2.5 (AB; blue) and high iron diamine (HID; brown) staining of goblet cells and Brunner’s glands were used to identify sialylated and sulfated acidic glycoproteins and to calculate the secretion rate (SSR) in young and adult mice fed ND, HFD, or HFD+Fru diets (Figure 4A,B). In young mice, Brunner’s glands and villus goblet cells showed a similar pattern, a statistically significant increase in SSR in both HFD and HFD+Fru groups compared with ND. Specifically, SSR increased by 25–26% in the glands and 17–19% in the villus goblet cells (Figure 4C). In adult mice, no appreciable change in SSR was detected in Brunner’s glands. By contrast, goblet cells of the duodenal villi displayed reduced AB pH 2.5 staining intensity in both HFD and HFD+Fru groups, with SSR showing a statistically significant decrease of approximately 5% compared with controls (Figure 4D).

#### 2.1.3. Lectin Histochemistry

##### SNA for the Identification of Sialic Acid

**Figure 5:**
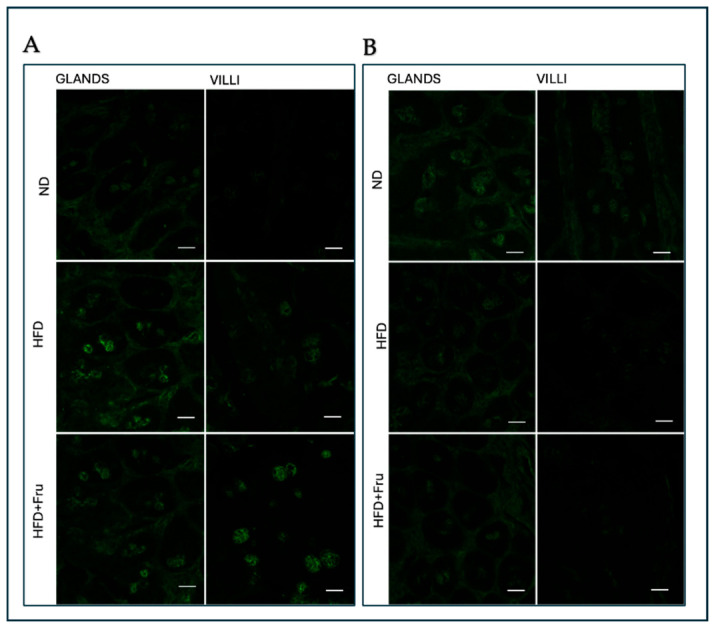
(**A**) Staining with SNA-FITC lectin in young mice to detect sialic acid in Brunner’s glands and villus goblet cells. Scale bar: 20 µm. (**B**) Staining with SNA-FITC lectin in adult mice to detect sialic acid in Brunner’s glands and villus goblet cells. Scale bar: 20 µm.

**Figure 6:**
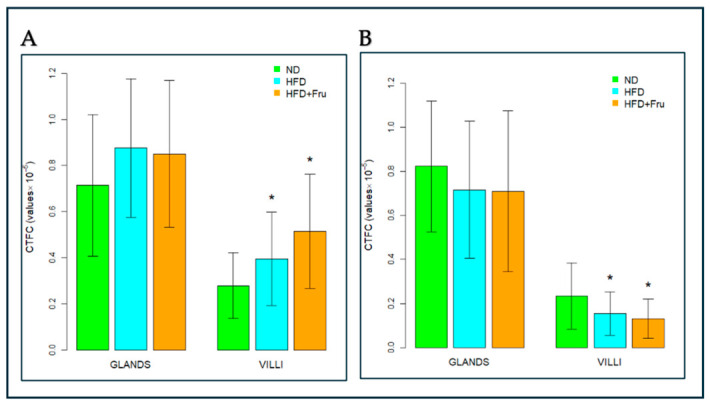
SNA-FITC lectin binding in Brunner’s glands and villus goblet cells of young and adult mice fed a normal diet (ND), high-fat diet (HFD), or high-fat diet supplemented with fructose (HFD+Fru): (**A**) Young mice. (**B**) Adult mice. SNA-FITC labeling was quantified to assess α2,6-linked sialic acid residues. Data for SNA-FITC lectin, presented as means ± SD, were analyzed by Kruskal–Wallis in both young mice (**A**) and adult mice (**B**). An asterisk (*) indicates a statistically significant difference from the ND group (*p*-value < 0.05).

In young mice, Brunner’s glands showed an increase in fluorescence signal in both HFD and HFD+Fru groups compared with ND; however, these differences were not statistically significant. In villus goblet cells, lectin fluorescence increased significantly by 30% in the HFD group and 46% in the HFD+Fru group compared to ND (Figure 5A and Figure 6A).

In adult mice, Brunner’s glands showed lower lectin fluorescence in both the HFD and HFD+Fru groups compared with ND, although these reductions were not statistically significant. In villus goblet cells, lectin positivity was significantly decreased by 35% in the HFD group and 43% in the HFD+Fru group relative to ND (Figure 5B and Figure 6B).

##### SBA for the Identification of N-Acetylgalactosamine

**Figure 7:**
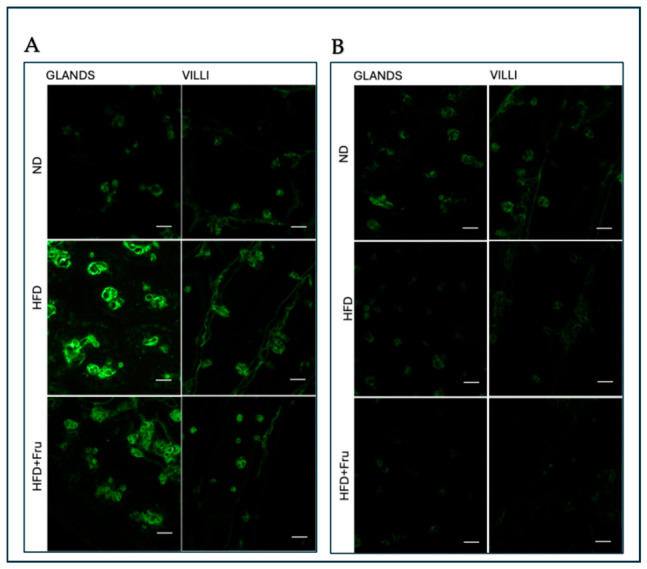
(**A**) Staining with SBA-FITC lectin in young mice to detect N-acetylgalactosamine in Brunner’s glands and villus goblet cells. Scale bar: 20 µm. (**B**) Staining with SBA-FITC lectin in adult mice to detect N-acetylgalactosamine in Brunner’s glands and villus goblet cells. Scale bar: 20 µm.

**Figure 8:**
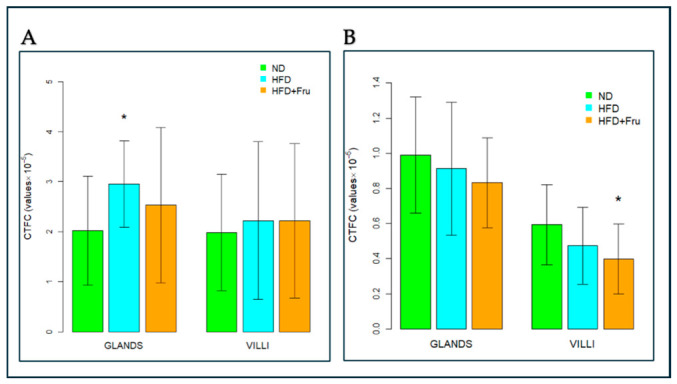
SBA-FITC lectin binding in Brunner’s glands and villus goblet cells of young and adult mice fed a normal diet (ND), high-fat diet (HFD), or high-fat diet supplemented with fructose (HFD+Fru): (**A**) Young mice. (**B**) Adult mice. SBA-FITC labeling was quantified to assess N-acetylgalactosamine residues. Data for SBA-FITC lectin, shown as means ± SD, were analyzed by the Kruskal–Wallis test for young mice (**A**) and one-way ANOVA for adult mice (**B**). An asterisk (*) indicates a statistically significant difference in mean value compared with the ND group (*p*-value < 0.05).

In young mice, Brunner’s glands showed a moderate increase in lectin positivity in the HFD and HFD+Fru groups compared with ND, although the latter difference was not statistically significant. In villus goblet cells, a slight increase in SBA staining was observed in the treated groups relative to ND, but this change was not statistically significant (Figure 7A and Figure 8A).

In adult mice, SBA positivity in Brunner’s glands was slightly reduced in both the HFD and HFD+Fru groups compared with ND, although this decrease was not statistically significant. In villus goblet cells, N-acetylgalactosamine staining was significantly reduced by 32% in the HFD+Fru group compared to the ND (Figure 7B and Figure 8B).

##### PNA for the Identification of Terminal Galactose β1,3N-Acetylgalactosamine

**Figure 9:**
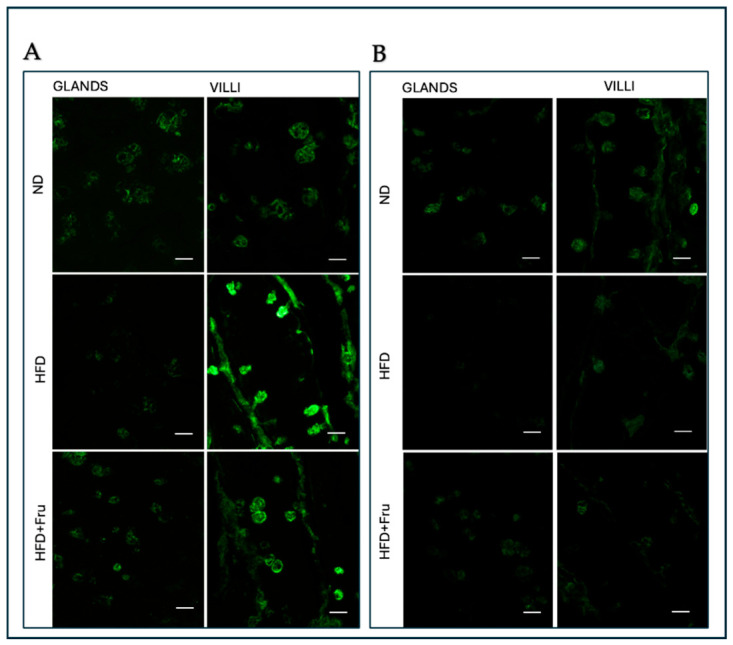
(**A**) Staining with PNA-FITC lectin in young mice to detect terminal Galactose β1,3N-acetylgalactosamine in Brunner’s glands and villus goblet cells. Scale bar: 20 µm. (**B**) Staining with PNA-FITC lectin in adult mice to detect terminal Galactose β1,3N-acetylgalactosamine in Brunner’s glands and villus goblet cells. Scale bar: 20 µm.

**Figure 10:**
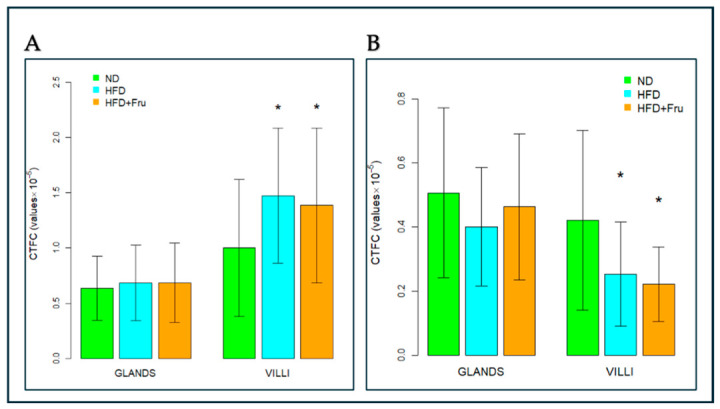
PNA-FITC lectin binding in Brunner’s glands and villus goblet cells of young and adult mice fed a normal diet (ND), high-fat diet (HFD), or high-fat diet supplemented with fructose (HFD+Fru): (**A**) Young mice. (**B**) Adult mice. PNA-FITC labeling was quantified to assess terminal Galβ1-3GalNAc residues. Data for PNA-FITC lectin, presented as means ± SD, were analyzed by the Kruskal–Wallis test in both young mice (**A**) and adult mice (**B**). An asterisk (*) indicates a statistically significant difference from the ND group (*p*-value < 0.05).

In young mice, Brunner’s glands showed a slight increase in PNA signal in the HFD and HFD+Fru groups compared with ND, but this was not statistically significant. In villus goblet cells, PNA fluorescence increased significantly by 47% in the HFD group and 39% in the HFD+Fru group compared to ND (Figure 9A and Figure 10A).

In adult mice, PNA fluorescence in Brunner’s glands was lower both in the HFD and HFD+Fru groups compared with ND, although this decrease was not statistically significant. In villus goblet cells, a similar pattern was observed, with reduced PNA signal in both the HFD and HFD+Fru groups; the reduction was statistically significant, amounting to 40% in the HFD group and 48% in the HFD+Fru group compared to ND (Figure 9B and Figure 10B).

##### WGA for the Identification of N-Acetylglucosamine

**Figure 11:**
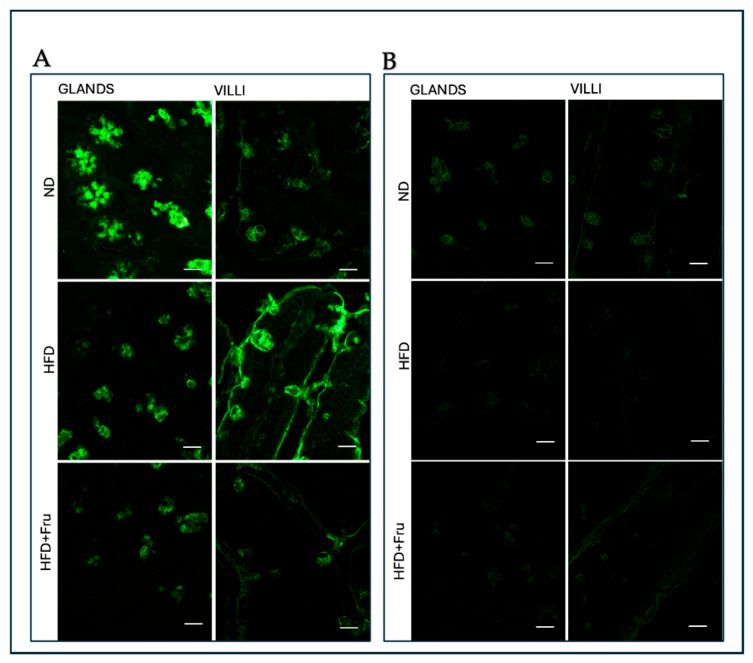
(**A**) Staining with WGA-FITC lectin in young mice to detect N-acetylglucosamine in Brunner’s glands and villi goblet cells. Scale bar: 20 µm. (**B**) Staining with WGA-FITC lectin in adult mice to detect N-acetylglucosamine in Brunner’s glands and villus goblet cells. Scale bar: 20 µm.

**Figure 12:**
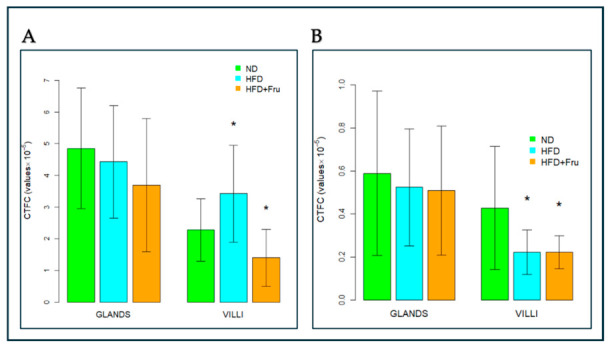
WGA-FITC lectin binding in Brunner’s glands and villus goblet cells of young and adult mice fed a normal diet (ND), high-fat diet (HFD), or high-fat diet supplemented with fructose (HFD+Fru): (**A**) Young mice. (**B**) Adult mice. WGA-FITC labeling was quantified to assess N-acetylglucosamine residues. Data for WGA-FITC lectin, shown as means ± SD, were analyzed by the Kruskal–Wallis test in both young mice (**A**) and adult mice (**B**). An asterisk (*) indicates a statistically significant difference in mean value compared with the ND group (*p*-value < 0.05).

In young mice, Brunner’s glands showed reduced lectin positivity in both HFD and HFD+Fru groups compared with ND, although this change was not statistically significant. In villus goblet cells, WGA fluorescence significantly doubled in the HFD group compared with ND, whereas it was significantly reduced in the HFD+Fru group compared to ND (Figure 11A and Figure 12A).

In adult mice, the WGA signal in Brunner’s glands was lower in both the HFD and HFD+Fru groups compared with ND, although these differences were not statistically significant. In villus goblet cells, lectin fluorescence was significantly halved in both the HFD and HFD+Fru groups compared with ND (Figure 11B and Figure 12B).

##### AAL for the Identification of α (1,6)L-Fucose

**Figure 13:**
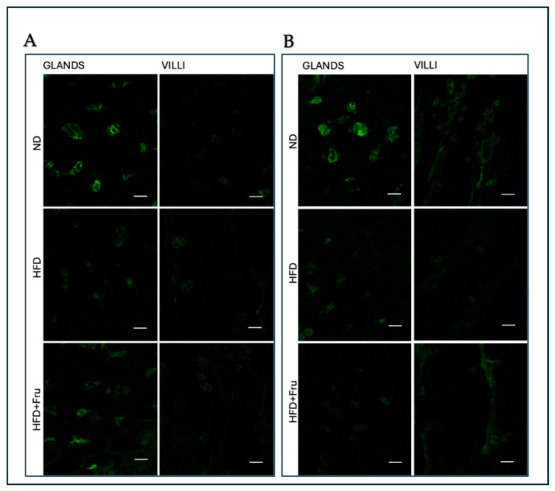
(**A**) Staining with AAL-FITC lectin in young mice to detect α (1,6)L-fucose in Brunner’s glands and villus goblet cells. Scale bar: 20 µm. (**B**) Staining with AAL-FITC lectin in adult mice to detect α (1,6)L-fucose in Brunner’s glands and villus goblet cells. Scale bar: 20 µm.

**Figure 14:**
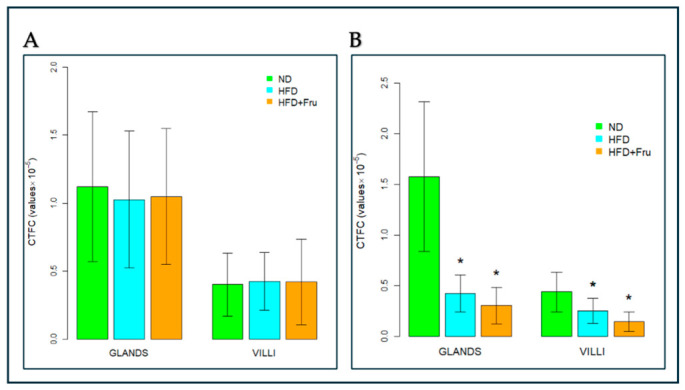
AAL-FITC lectin binding in Brunner’s glands and villus goblet cells of young and adult mice fed a normal diet (ND), high-fat diet (HFD), or high-fat diet supplemented with fructose (HFD+Fru): (**A**) Young mice. (**B**) Adult mice. AAL-FITC labeling was quantified to assess fucosylated glycans. Data for AAL-FITC lectin, presented as means ± SD, were analyzed by the Kruskal–Wallis test in both young mice (**A**) and adult mice (**B**). An asterisk (*) indicates a statistically significant difference from the ND group (*p*-value < 0.05).

In young mice, Brunner’s glands showed a decrease in fluorescence signal in both the HFD and HFD+Fru groups compared with ND, although this change was not statistically significant. A similar, non-significant trend was observed in villus goblet cells (Figure 13A and Figure 14A).

In adult mice, Brunner’s glands showed a statistically significant reduction in AAL fluorescence of 73% in the HFD group and 81% in the HFD+Fru group compared with the ND group. A similar pattern was observed in villus goblet cells, with significant reductions of 43% in the HFD group and 66% in the HFD+Fru group compared to ND (Figure 13B and Figure 14B).

## 3. Discussion

In the small intestine, the primary site of nutrient absorption, fructose induces age-specific responses: villi elongation in young animals (enhancing absorptive capacities) and vacuolization with atrophy in adults [15]. Duodenal mucus is primarily secreted by villus goblet cells (Muc2) and Brunner’s glands (Muc6), which together form the protective mucus barrier. The present study investigates the interaction between an HFD, fructose intake, and intestinal health, focusing on age-dependent alterations in mucin glycosylation profiles in Brunner’s glands, goblet cells, and duodenal villi. In this context, the effects observed should be interpreted as resulting from fructose supplementation within an HFD background, rather than reflecting the isolated action of fructose per se. Our results support the hypothesis that the addition of fructose to an HFD induces distinct alterations in the mucus layer, with age-dependent responses between young and adult mice. The age-dependent differences observed in this study can be interpreted within the broader framework of intestinal aging. The intestinal epithelium is a highly dynamic tissue characterized by rapid turnover and continuous regeneration, processes that are progressively altered with age. In younger individuals, high epithelial plasticity, an efficient unfolded protein response (UPR), and robust goblet cell function support adaptive responses to metabolic stress, including increased mucin production and remodeling. In contrast, more mature stages are associated with reduced regenerative capacity, impaired proteostasis, and increased susceptibility to chronic endoplasmic reticulum (ER) stress, all of which can compromise mucin biosynthesis and secretion. In addition, age-related alterations in host–microbiota interactions and low-grade inflammation further contribute to mucus barrier dysfunction [11]. Within this framework, our findings indicate that the response is characterized by a compensatory phenotype in young mice and a maladaptive phenotype in adult mice. Consistent with previous nutritional models, both HFD and HFD+Fru resulted in a significant increase in body weight, although the magnitude of the effect differed between age groups. Young mice exhibited the greatest weight gain on HFD, whereas HFD+Fru produced an intermediate increase. This suggests that lipid content primarily drives diet-induced obesity, with fructose acting mainly as a modulator in the absence of additional risk factors [16,17]. In adult mice, both diets markedly increased body weight relative to ND, with the highest values observed in the HFD+Fru group. This aligns with studies highlighting increased vulnerability to high-fat and high-sugar diets during advanced developmental stages and age-dependent effects on lipid metabolism [17]. The differing responses of villus goblet cells to HFD+Fru in young versus adult mice reflect their distinct capacities to cope with diet-induced metabolic stress. In young animals, the increase in PAS positivity indicates a compensatory response, where controlled activation of the UPR supports the overproduction of protective glycoproteins [18,19]. By contrast, the marked decrease in PAS staining in adult mice indicates a failure of this adaptive mechanism. According to previous studies, the synergistic action of fat and fructose may be associated with chronic and dysfunctional endoplasmic reticulum (ER) stress, where the accumulation of misfolded mucins may activate the pro-apoptotic PERK–CHOP pathway, inhibit protein synthesis, and compromise the secretory capacity of the villus epithelium [20]. While Brunner’s glands maintain relative structural and functional stability, the specific vulnerability of duodenal villi in adult mice suggests that aging diminishes the villus epithelium’s ability to manage the proteotoxic load from the diet, predisposing it to functional collapse of the mucosal barrier [21,22]. Conventional histochemical methods confirmed the presence of both sialylated and sulfated mucins in young and adult mice, revealing distinct age-dependent patterns in the sialo-sulfomucin ratio (SSR) in response to HFD+Fru [7]. In young mice, the increase in SSR in Brunner’s glands and villus goblet cells reflects a shift toward more sialylated and relatively less sulfated mucins, consistent with extensive duodenal mucin remodeling due to both diets. This reduction in sulfated mucins may weaken the mucus layer, making it more susceptible to bacterial degradation and potentially favoring local inflammation [23]. Conversely, in the duodenal villi of adult mice, the decrease in SSR, coupled with stable patterns in Brunner’s glands, suggests greater mucin sulfation. Inflammatory signals from HFD+Fru likely promote the upregulation of sulfotransferases, enhancing mucin sulfation and leading to mucosal alterations, impaired tight junctions, and increased intestinal permeability, as reported in HFD models with compromised barrier integrity [24]. Lectin histochemistry revealed diet and age-dependent changes in goblet cell mucin glycosylation. In young mice, enhanced SNA signals for α2,6-linked sialic acid in villus goblet cells indicate increased mucin sialylation, consistent with the elevated SSR [25]. Beyond local defense, altered sialylation has broader implications: increased surface sialylation is a recognized hallmark in tumor biology and is often associated with immune evasion through interaction with inhibitory Siglec receptors [5,26,27]. While neoplastic lesions were not observed in this model, the increased sialic acid content in young animals may indicate a mucosal sialylation profile that, if chronically maintained alongside other predisposing factors, could contribute to a microenvironment permissive for neoplastic transformation [28]. The observed reduction in mucin sialylation is particularly significant, as sialic acid residues are crucial for the viscoelastic and protective properties of mucus; their decrease weakens the intestinal barrier, exposing the duodenal epithelium to luminal stressors and promoting intestinal permeability and inflammation [29]. The analysis of GalNAc-containing glycans using SBA underscores this age-dependent divergence. In adult mice fed HFD+Fru, a marked reduction in SBA binding was observed in villus goblet cells. Alongside decreased SSR and an increased proportion of sulfated mucins, this pattern may suggest an accumulation of sulfated O-glycans that can sterically mask terminal GalNAc residues recognized by SBA. HFD+Fru-induced dysbiosis is expected to enhance bacterial glycosidase activity, further depleting exposed GalNAc epitopes [30]. This combination of host-driven hypersulfation and microbiota-mediated glycan degradation results in a mucosal barrier that is heavily remodeled yet functionally compromised. In addition, PNA lectin, which specifically recognizes Galβ1–3GalNAc (Thomsen–Friedenreich (TF) antigen), provides further insight into the status of mucin O-glycosylation [7,31]. In our study, increased PNA reactivity was primarily observed in young mice, suggesting enhanced exposure of these core 1 structures and indicating incomplete or truncated O-glycosylation. This phenomenon is well documented in the literature and is commonly associated with alterations in glycosylation pathways under conditions of metabolic or inflammatory stress [31]. The exposure of TF antigen suggests a shift toward shorter O-glycan chains, which may influence the structural organization and physicochemical properties of mucins [31]. In particular, truncated glycosylation has been associated with a reduced gel-forming capacity of MUC2, potentially resulting in a less organized mucus architecture. These findings reflect a diet- and age-dependent remodeling of mucin glycosylation. In young mice, the increase in terminal Gal suggests altered mucus properties, where metabolic stress may inhibit sulfotransferase activity, leaving mucins largely unsulfated and therefore more accessible to bacterial glycosidases [32]. In adult mice, the reduction in galactose aligns with an inflammation-driven adaptive mechanism, where sustained activation of sulfotransferases enhances incorporation of sulfate groups into mucins, masking key binding sites and increasing resistance to the pro-inflammatory luminal environment [32]. The increase in sulfate groups in adult HFD+Fru mice likely represents an initial protective response to chronic inflammation and dysbiosis, which, under prolonged stress and age-related fragility, becomes maladaptive. This shift generates a more acidic mucus with altered viscoelastic properties and reduced protective capacity, creating a milieu conducive to further disruption of the intestinal barrier [33,34,35,36]. GlcNAc is another key component of intestinal mucus, contributing to the formation of a protective apical glycocalyx and limiting direct bacterial contact with the epithelium [37]. In young mice fed HFD+Fru, the marked reduction in WGA binding in duodenal villi coincided with increased SNA reactivity in goblet cells, which may indicate a shift toward more highly sialylated O-glycans. In a fructose-rich, pro-inflammatory environment, enhanced sialylation may divert glycan maturation toward Neu5Ac-terminated structures, effectively depleting high-affinity GlcNAc epitopes for WGA and eroding a key component of the protective glycocalyx [38]. In adult mice, the combined reduction in WGA binding, decreased sialylation, and increased sulfated mucins suggest that chronic HFD+Fru is associated with remodeling of GlcNAc-containing glycans toward a more sulfated mucin profile, compatible with a barrier less enriched in WGA-detectable GlcNAc motifs but better adapted to withstand chronic inflammatory and microbial stress [34]. Finally, AAL lectin staining revealed a significant age- and diet-dependent decrease in fucose expression in both the duodenal villi of adult mice and Brunner’s glands, particularly under HFD+Fru. Fucose plays a key role in cell adhesion and immune modulation, acting as a terminal residue on intestinal mucins such as MUC2 and as an essential mediator of host–microbe interactions [39]. Its reduction in the Western diet, as reported in murine models at the level of duodenal mucins, suggests that dietary habits may affect both metabolic health and the structural and immunological integrity of the intestinal mucosa [40]. Altered fucosylation patterns may therefore contribute to barrier dysfunction, dysbiosis, increased permeability, and low-grade systemic inflammation, all of which are strongly associated with obesity, metabolic dysfunction-associated steatotic liver disease (MASLD), and related metabolic disorders [41,42].

Our study presents several limitations. The first limitation is the exclusive use of male mice. Sex-specific differences in metabolic regulation and intestinal physiology have been widely reported and may influence both mucin production and glycosylation patterns. In particular, sex hormones such as estrogens have been shown to modulate epithelial barrier integrity, immune responses, and host–microbiota interactions, all of which are key determinants of mucus composition and function [43,44]. Therefore, the absence of female cohorts prevents us from addressing potential sex-dependent variability in the remodeling of duodenal mucin glycosylation induced by fructose-rich diets. While the use of male mice allowed us to reduce variability associated with hormonal fluctuations and to better isolate diet- and age-dependent mechanisms, future studies including both sexes will be necessary to validate and extend the translational relevance of these findings.

The second limitation of the present study is the lack of baseline and longitudinal measurements. Although the animals were age-matched and randomly assigned to experimental groups under controlled conditions, the absence of time-course data prevents the assessment of the temporal progression of diet-induced changes in mucin production and glycosylation. Given that our analysis focused on endpoint histological and glycohistochemical outcomes following prolonged dietary exposure, this approach allowed the identification of stable mucosal remodeling.

Another limitation is the lack of a group fed a high-fructose diet alone, enabling us to distinguish the effects of fructose from some of the potential synergistic effects with HFD. On the other hand, most of the published studies on high-fructose diets, comparable with our content of fructose (30% of calories), do not describe the remaining nutrient composition of the diet, whereas others used fructose in addition to an HFD similar to our experimental design [45], since the metabolic mechanisms related to fructose are distinct from fats and other sugars [46]. On the other hand, we aimed to mimic the Western diet rich in saturated fats and fructose, although we used a percentage of fructose that is standard for mice but quite higher than that reported in literature in human studies [47]. Future studies incorporating longitudinal analyses and a diet enriched in fructose alone and also with a lower percentage of free fructose will be essential to better define the kinetics of intestinal adaptation and barrier dysfunction in response to high-fat and fructose-rich diets.

Finally, the relatively small sample size limited the application of a unified factorial statistical approach, such as two-way ANOVA, to simultaneously assess the effects of diet, age, and their interaction. In particular, the estimation of interaction effects would benefit from a larger number of animals per group. Future studies involving larger cohorts will allow for the implementation of a more comprehensive statistical framework, thereby strengthening the analysis of diet- and age-dependent effects.

## 4. Materials and Methods

### 4.1. Animals

Wild-type (WT) male C57BL/6 mice were purchased from Charles River Laboratories (Wilmington, MA, USA) and maintained under specific pathogen-free conditions. Mice were housed under standard animal facility conditions in a temperature-controlled room (RT) (22 ± 2 °C) under a 12 h light/12 h dark cycle, with free access to food and water. Three-week-old (young) or six-week-old (adult) mice were fed an HFD for 16 weeks, either alone or supplemented with 30% (*w*/*v*) fructose in the drinking water. The high-fat diet consisted of a standard purified rodent diet providing 60% of total energy from fat (20% carbohydrate and 20% protein), with fat primarily derived from lard and soybean oil. The diet and fructose were commercially obtained from Laboratorio Dottori Piccioni (Gessate, Italy). Control animals received a standard diet and regular drinking water throughout the study. At the end of the treatment period, mice were anesthetized via intraperitoneal injection of a Zoletil/Xylazine mixture (43 mg/kg and 17.2 mg/kg, respectively). After confirmation of adequate anesthesia, blood samples were collected by cardiac puncture. Animals were then euthanized by cervical dislocation, and tissues were harvested and cryopreserved for subsequent analyses. All animal procedures were performed at the animal facility of the Department of Health Sciences, University of Eastern Piedmont (Novara, Italy), in accordance with European Union guidelines for the care and use of laboratory animals. The experimental protocols were approved by the Italian Ministry of Health (Authorization No. 893/2023-PR).

### 4.2. Section Preparation

Thirty mice (15 young and 15 adults) were divided into three dietary conditions (ND, HFD, and HFD+Fru). From each condition, duodenal samples were collected from five young and five adult mice and immediately fixed in 4% paraformaldehyde overnight at 4 °C. Then, the samples were incubated in 15% sucrose in PBS at 4 °C. The duodenal samples were dehydrated in a graded series of ethanol and embedded in paraffin wax. Sections were cut at 5 µm thickness.

### 4.3. Classical Histochemical Staining

To perform histochemical investigations, in both young and adult mice, the sections were dewaxed and rehydrated in a graded series of ethanol. The duodenal sections were stained with Periodic Acid–Schiff (PAS) to demonstrate the presence of polysaccharides, glycoproteins, and other substances containing glycolic or aminohydroxyl groups in tissue samples. The acid groups were identified by two different histochemical methods performed separately: to highlight sialylated glycoproteins, the sections were stained with AB pH 2.5 for 30 min; to detect sulfated ones, HID, a solution containing N,N-dimethylmetaphenylene diamine dihydrochloride, N,N-dimethylparaphenylene diamine dihydrochloride, and freshly prepared ferric chloride (60% *w*/*v*), was used for 18 h. The sections were dehydrated and mounted with DPX. Reagents were sourced from Sigma-Aldrich Inc. (St. Louis, MO, USA).

### 4.4. Lectins

Five lectins conjugated to FITC (PNA, SBA, WGA, AAL, and SNA) were used to characterize possible alterations at the level of individual oligosaccharide residues of glycoproteins in young and adult mice. Lectins were sourced from Vector Laboratories, Newark, CA, USA. The common names, sugar specificities, and concentrations of the lectins used are shown in Table 2. Sections of young and adult mice were rehydrated in a series of ethanol, and they were incubated for 1 h at RT, with lectins diluted in HEPES pH 7.4, according to the manufacturer’s instructions. The slides were washed with the same dilution buffer and mounted with Fluoromount. For details, see Mentino et al. [48] and Mastrodonato et al. [7]. The controls implemented for lectin histochemistry included (1) substituting the lectin solution with buffer alone; (2) preincubating the tissue sections with the specific hapten sugar inhibitor at a concentration of 0.2 M; and (3) testing binding on samples known to contain mucins previously demonstrated to be labeled by the lectins used in this study [49,50,51].

### 4.5. Quantitative Analysis

Images were captured using a Nikon Eclipse Ni epifluorescence microscope and a DS-Fi3 digital camera (Nikon Instruments Ltd., Campi Bisenzio, FI, Italy) under the same conditions for all samples. PAS, AB 2.5, and HID were observed in bright light, whereas lectins were observed in epifluorescence under 495 nm light emission. For histochemical staining, the images were acquired at 20× magnification. An analysis of the variation in the intensity of each stain between ND, HFD, and HFD+Fru groups was performed by considering ten Brunner’s glands and ten duodenal goblet cells for each mouse (young and adult) and each diet group (ND, HFD, and HFD+Fru), with the best orientation selected in each photo. Analyses were performed by the ImageJ package (Version 2), and the histochemical staining intensities were estimated by computing integrated optical density using the formula OD = log (255/mean intensity). For lectin fluorescence staining, the images were acquired at 20× magnification, and an analysis of the variation in the intensity of each lectin-binding between ND, HFD, and HFD+Fru groups was performed for the same number of Brunner’s glands and duodenal goblet cells. For lectin-binding intensities, the Corrected Total Cell Fluorescence (CTCF) for each gland area and cell was computed.

### 4.6. Statistical Analysis

The statistical analysis was performed to determine whether there were significant differences among the group means. To avoid pseudoreplication, the animal was considered the experimental unit. For quantitative analyses, measurements obtained from multiple glands and cells within each animal were averaged to generate a single value per mouse, and statistical analyses were performed using these animal-level mean values.

Before conducting inferential testing, data distribution assumptions were evaluated. The Shapiro–Wilk test was used to assess the normality of model residuals, and the homogeneity of variances was evaluated using Bartlett’s test. Based on these diagnostic results, for data meeting the assumptions of normality and homoscedasticity, a one-way analysis of variance (ANOVA) was performed. This was followed by Tukey’s Honestly Significant Difference post hoc test for all pairwise comparisons. In cases of unequal variances (heteroscedasticity) with normally distributed data, Welch’s ANOVA was applied, followed by the Games–Howell post hoc test. When the assumption of normality was violated, the non-parametric Kruskal–Wallis test was employed, followed by Dunn’s test with Bonferroni correction for multiple comparisons. The results of the statistical tests are shown in Tables S1–S6 in the Supplementary Materials. Data are presented as mean ± standard deviation (SD), and an asterisk (*) denotes a statistically significant difference in the mean value compared with the ND group (*p*-value < 0.05). All analyses were conducted using R software (Version 4.5.1).

## 5. Conclusions

In conclusion, this study demonstrates that fructose supplementation in the context of a high-fat diet results in an age-dependent remodeling of duodenal mucin glycosylation, with differential effects on Brunner’s glands and villus goblet cells. In young mice, the diet induces a still plastic and compensatory mucus response, characterized by increased PAS reactivity, increased sialylation, and reduced sulfation. While this profile may favor short-term barrier maintenance, prolonged exposure could promote the appearance of an altered glycan signature. In adult mice, however, diet reveals age-related villous vulnerability: the loss of PAS-positive mucins, hypersulfation, reduced sialylation, and fucosylation converge toward more acidic and functionally altered mucus. These findings emphasize that diet-induced alterations cannot be fully understood without considering both age and the regulation of mucin O-glycosylation as key determinants of mucus layer integrity. Future studies should investigate the cellular mechanisms underlying these alterations in more detail, with particular attention to intercellular junction proteins such as occludin and zonulin. Further ultrastructural analyses will clarify how these interactions functionally contribute to intestinal homeostasis.

## Figures and Tables

**Table 1:** Statistical comparisons of body weight values among the ND, HFD, and HFD+Fru groups performed in both young and adult mice.

WEIGHTS	Group	Mean ± SD	Median (IQR)	F (*p*-Value)	SW (*p*-Value)	B (*p*-Value)	*p*-Values from Post Hoc Tests
Young	ND	28.09 ± 1.33	27.95 (2.33)	${17.11\;}^{W}$ (0.0002 *)	0.943 (0.107)	9.869 (0.007 *)	0.001 * (HFD); 0.008 * (HFD+Fru)
	HFD	35.21 ± 4.25	35.10 (4.38)				0.321 (HFD+Fru)
	HFD+Fru	32.59 ± 3.60	32.55 (4.18)				
Adult	ND HFD	34.72 ± 3.77 39.93 ± 4.58	33.95 (4.78) 39.67 (2.88)	8.617 (0.001 *)	0.972 (0.602)	0.340 (0.844)	0.028 * (HFD); 0.001 * (HFD+Fru) 0.394 (HFD+Fru)
	HFD+Fru	42.45 ± 4.35	42.65 (5.70)				

Abbreviations: SD, standard deviation; IQR, interquartile range; SW, Shapiro–Wilk test for normality of distribution; B, Bartlett’s test for homogeneity of variances; F, ANOVA test; *^W^*, Welch ANOVA. The asterisk indicates that the test was statistically significant (*p*-value < 0.05).

**Table 2:** Lectins used, their carbohydrate specificities, and related inhibitory sugars in control experiments.

Lectin	Source	Binding Specificity	Lectin Concentration (mg/mL)	Inhibitory Sugar
PNA	*Arachis hypogaea*	Galβ1,3GalNAc	10 mg/mL	0.2 M Gal
SBA	*Glycine max*	GalNAc/Gal	20 mg/mL	0.2 M GalNAc
WGA	*Triticum wulgaris*	(GlcNAcβ1,4)n	20 mg/mL	0.5 M GlcNac
SNA	*Sambucus nigra*	Neu5Ac(α2-6)Gal	10 mg/mL	0.2 M Lac
AAL	*Aleuria aurantia*	Fucα1,6GlcNAcβNAsn; Fucα1,3,Fucα1,4	10 mg/mL	0.2 M L-Fuc

Abbreviations: Fuc, fucose; Gal, galactose; GalNac, N-acetylgalactosamine; GlcNAc, N-acetylglucosamine; Neu5Ac, N-acetylneuraminic acid [52,53,54,55].

## Data Availability

The original contributions presented in this study are included in the article/Supplementary Material. Further inquiries can be directed to the corresponding author.

## Supplementary Materials

The following supporting information can be downloaded at: https://www.mdpi.com/article/10.3390/ijms27104189/s1.

## Abbreviations

The following abbreviations are used in this manuscript:

HFCS	High-fructose corn syrup
Muc2	Mucin 2
Muc6	Mucin 6
ND	Normal diet
HFD	High-fat diet
HFD+Fru	High-fat diet supplemented with fructose
SD	Standard deviation
IQR	Interquartile range
SW	Shapiro–Wilk test for normality of distribution
B	Barlett’s test for homogeneity of variances
F	ANOVA test
T	Turkey’s test for multiple comparisons of means
W	Welch ANOVA followed by Games–Howell test for multiple comparisons of means
D	Dunnett’s test for multiple comparisons of means with the ND group
PAS	Periodic Acid–Schiff
AB pH 2.5	Alcian Blue pH 2.5
HID	High iron diamine
SSR	Sialo-sulfomucin ratio
SNA	*Sambucus nigra*
SBA	*Glycine max*
PNA	*Arachis hypogaea*
WGA	*Triticum vulgaris*
AAL	*Aleuria aurantia*
UPR	Unfolded protein response
ER	Endoplasmic reticulum
PERK–CHOP	Protein Kinase RNA-Like Endoplasmic Reticulum Kinase, C/EBP Homologous Protein
GalNAc	N-acetylgalactosamine
Gal	Galactose
Neu5Ac	N-acetylneuraminic acid
WT	Wild type
DPX	Dibutylphthalate polystyrene xylene
RT	Room temperature
Lac	Lactose
Fuc	Fucose
H&E	Hemalum–eosin
CTCF	Corrected total cell fluorescence
CTRL	Control
