# Supplementary material for: Dietary Fructose Alters Duodenal Mucin Glycosylation and Mucus Production in High-Fat Diet-Fed Mice
Source: Int J Mol Sci. 2026 May 8;27(10):4189. doi: 10.3390/ijms27104189 (PMC13207898; doi:10.3390/ijms27104189)
Supplement: Supplementary file 1 [file ijms-27-04189-s001.zip › ijms-4197859-supplementary.pdf]

Table S1. Statistical comparisons of Glands and Villi CTFC × 10^{-5} values between normal diet (ND), high-fat diet (HFD), and HFD+Fru groups in young mice.

LECTIN	Group	Mean ± SD	Median (IQR)	KW (p-value)	SW (p-value)	B (p-value)	p-values from post hoc tests
**WGA** Glands	ND	5.24 ± 1.98	4.77 (8.03)	5.343 (0.069)	0.917 (3e-05*)	0.844 (0.656)	-
	HFD	4.43 ± 1.78	3.58 (4.91)				
	HFD+Fru	3.53 ± 2.20	3.20 (6.30)				
**WGA** Villi	ND	2.28 ± 0.98	1.80 (3.42)	30.948 (2e-07*)	0.946 (0.001*)	9.632 (0.008*)	0.013* (HFD); 0.022* (HFD+Fru)
	HFD	3.43 ± 1.53	3.30 (5.03)				8e-08* (HFD+Fru)
	HFD+Fru	1.40 ± 0.90	1.35 (2.73)				
**SBA** Glands	ND	2.02 ± 1.09	2.16 (3.49)	9.668 (0.008*)	0.939 (0.0004*)	9.941 (0.007*)	0.002* (HFD); 0.221 (HFD+Fru)
	HFD	2.95 ± 0.87	3.51 (2.59)				0.062 (HFD+Fru)
	HFD+Fru	2.53 ± 1.55	1.88 (4.44)				
**SBA** Villi	ND	1.98 ± 1.16	1.98 (3.91)	0.809 (0.667)	0.863 (1e-07*)	2.958 (0.228)	-
	HFD	2.22 ± 1.58	1.55 (3.98)				
	HFD+Fru	2.22 ± 1.54	1.88 (4.94)				
**SNA** Glands	ND	0.72 ± 0.31	0.76 (0.96)	2.708 (0.258)	0.966 (0.017*)	0.094 (0.954)	-
	HFD	0.88 ± 0.30	0.82 (0.83)				
	HFD+Fru	0.85 ± 0.32	0.79 (1.18)				
**SNA** Villi	ND	0.28 ± 0.14	0.27 (0.48)	17.073 (2e-04*)	0.902 (5e-06*)	8.116 (0.017*)	0.049* (HFD); 4e-05* (HFD+Fru)
	HFD	0.40 ± 0.20	0.32 (0.83)				0.031* (HFD+Fru)
	HFD+Fru	0.52 ± 0.25	0.39 (0.73)				
**PNA** Glands	ND	0.64 ± 0.29	0.58 (0.97)	1.233 (0.890)	0.949 (0.001*)	1.413 (0.494)	-
	HFD	0.68 ± 0.34	0.52 (1.21)				
	HFD+Fru	0.68 ± 0.36	0.67 (1.49)				
**PNA** Villi	ND	1.00 ± 0.62	0.74 (2.05)	11.104 (0.004*)	0.911 (1e-05*)	0.673 (0.714)	0.002* (HFD); 0.013* (HFD+Fru)
	HFD	1.47 ± 0.61	1.32 (1.93)				0.498 (HFD+Fru)
	HFD+Fru	1.39 ± 0.70	1.36 (2.08)				
**AAA** Glands	ND	1.12 ± 0.55	1.12 (1.46)	0.414 (0.813)	0.965 (0.017*)	0.344 (0.842)	-
	HFD	1.03 ± 0.50	0.89 (1.71)				
	HFD+Fru	1.05 ± 0.50	1.12 (2.02)				
**AAA** Villi	ND	0.40 ± 0.23	0.37 (0.82)	1.088 (0.580)	0.897 (3e-06*)	5.105 (0.078)	-
	HFD	0.42 ± 0.21	0.37 (0.74)				
	HFD+Fru	0.42 ± 0.32	0.23 (0.77)				

* Abbreviations: CTFC, corrected total cell fluorescence; SD, standard deviation; IQR, interquartile range; SW, Shapiro-Wilk test for normality of distribution; B, Bartlett's test for homogeneity of variances; KW, Kruskal-Wallis rank sum test. *The test was statistically significant (p-value < 0.05).

Table S2. Statistical comparisons of Glands and Villi SSR values between normal diet (ND), high-fat diet (HFD), and HFD+Fru groups in young mice.

SSR	Group	Mean ± SD	Median (IQR)	F (p-value)	KW (p-value)	SW (p-value)	B (p-value)	p-values from post hoc tests
Glands	ND	0.81 ± 0.06	0.81 (0.24)	247.3^{W} (2e-16*)	-	0.973 (0.126)	8.507 (0.014*)	8e-19* (HFD); 8e-13* (HFD+Fru)
	HFD	1.09 ± 0.05	1.08 (0.19)					0.753 (HFD+Fru)
	HFD+Fru	1.08 ± 0.03	1.08 (0.11)					
Villi	ND	0.87 ± 0.04	0.88 (0.18)	-	47.936 (4e-11*)	0.950 (0.011*)	6.256 (0.044*)	2e-08* (HFD); 6e-09* (HFD+Fru)
	HFD	1.08 ± 0.02	1.08 (0.08)					0.176 (HFD+Fru)
	HFD+Fru	1.05 ± 0.03	1.05 (0.10)					

* Abbreviations: SSR, secretion rate; SD, standard deviation; IQR, interquartile range; SW, Shapiro-Wilk test for normality of distribution; B, Bartlett's test for homogeneity of variances; F, ANOVA test; W, Welch ANOVA; KW, Kruskal-Wallis rank sum test. *The test was statistically significant (p-value < 0.05).

Table S3. Statistical comparisons of Glands and Villi PAS values between normal diet (ND), high-fat diet (HFD), and HFD+Fru groups in young mice.

PAS	Group	Mean ± SD	Median (IQR)	F (p-value)	SW (p-value)	B (p-value)	p-values from post hoc tests
Glands	ND	1.87 ± 0.06	1.87 (0.23)	29.531^{W} (2e-09*)	0.985 (0.415)	9.459 (0.009*)	4e-09* (HFD); 0.002* (HFD+Fru)
	HFD	1.71 ± 0.10	1.70 (0.35)				2e-04* (HFD+Fru)
	HFD+Fru	1.81 ± 0.07	1.82 (0.25)				
Villi	ND	1.96 ± 0.06	1.94 (0.24)	25.540 (2e-09*)	0.982 (0.283)	0.312 (0.855)	4e-05*(HFD); 0.023* (HFD+Fru)
	HFD	1.89 ± 0.06	1.88 (0.22)				< 1e-10* (HFD+Fru)
	HFD+Fru	2.00 ± 0.06	2.02 (0.20)				

* Abbreviations: PAS, periodic acid-Schiff; SD, standard deviation; IQR, interquartile range; SW, Shapiro-Wilk test for normality of distribution; B, Bartlett's test for homogeneity of variances; F, ANOVA test; W, Welch ANOVA. *The test was statistically significant (p-value < 0.05).

Table S4. Statistical comparisons of Glands and Villi CTFC × 10^{-5} values between normal diet (ND), high-fat diet (HFD), and HFD+Fru groups in adult mice.

LECTIN	Group	Mean ± SD	Median (IQR)	F (p-value)	KW (p-value)	SW (p-value)	B (p-value)	p-values from post hoc tests
**WGA** Glands	ND	0.59 ± 0.38	0.55 (0.46)	-	0.500 (0.780)	0.891 (1e-06*)	3.625 (0.163)	-
	HFD	0.52 ± 0.27	0.47 (0.48)					
	HFD+Fru	0.51 ± 0.30	0.49 (0.28)					
**WGA** Villi	ND	0.43 ± 0.29	0.44 (0.44)	-	9.250 (0.009*)	0.938 (0.0003*)	54.956 (1e-12*)	0.021* (HFD); 0.030* (HFD+Fru)
	HFD	0.22 ± 0.10	0.14 (0.14)					1 (HFD+Fru)
	HFD+Fru	0.22 ± 0.08	0.21 (0.09)					
**SBA** Glands	ND	0.99 ± 0.33	1.05 (0.39)	1.676 (0.193)	-	0.989 (0.697)	3.994 (0.135)	-
	HFD	0.91 ± 0.38	0.83 (0.59)					
	HFD+Fru	0.83 ± 0.26	0.83 (0.35)					
**SBA** Villi	ND	0.59 ± 0.23	0.54 (0.30)	-	10.433 (0.005*)	0.967 (0.023*)	0.543 (0.762)	0.168 (HFD); 0.004* (HFD+Fru)
	HFD	0.47 ± 0.22	0.46 (0.30)					0.566 (HFD+Fru)
	HFD+Fru	0.40 ± 0.20	0.38 (0.29)					
**SNA** Glands	ND	0.82 ± 0.30	0.85 (1.09)	-	4.21 (0.121)	0.937 (0.0003*)	1.407 (0.495)	-
	HFD	0.72 ± 0.31	0.74 (1.21)					
	HFD+Fru	0.71 ± 0.37	0.67 (1.21)					
**SNA** Villi	ND	0.23 ± 0.15	0.17 (0.50)	-	10.69 (0.005*)	0.907 (8e-06*)	9.318 (0.009)	0.042* (HFD); 0.005* (HFD+Fru)
	HFD	0.15 ± 0.10	0.12 (0.35)					1 (HFD+Fru)
	HFD+Fru	0.13 ± 0.09	0.11 (0.37)					
**PNA** Glands	ND	0.51 ± 0.26	0.51 (0.43)	-	2.275 (0.321)	0.951 (0.003*)	3.114 (0.211)	-
	HFD	0.40 ± 0.19	0.43 (0.34)					
	HFD+Fru	0.46 ± 0.23	0.48 (0.36)					
**PNA** Villi	ND	0.42 ± 0.28	0.74 (2.05)	-	8.577 (0.0137*)	0.951 (0.0021*)	22.498 (1e-05*)	0.033* (HFD); 0.035* (HFD+Fru)
	HFD	0.25 ± 0.16	0.26 (0.28)					1 (HFD+Fru)
	HFD+Fru	0.22 ± 0.12	0.16 (0.19)					
**AAA** Glands	ND	1.58 ± 0.74	0.31 (0.45)	-	54.713 (1e-12*)	0.712 (5e-12*)	59.337 (1e-13*)	2e-06* (HFD); 1e-12* (HFD+Fru)
	HFD	0.42 ± 0.18	0.43 (0.25)					7e-02* (HFD+Fru)
	HFD+Fru	0.30 ± 0.18	0.25 (0.26)					
**AAA** Villi	ND	0.44 ± 0.20	0.45 (0.40)	-	40.925 (1e-09*)	0.949 (0.0016*)	15.411 (0.0005*)	9e-03* (HFD); 5e-10* (HFD+Fru)
	HFD	0.25 ± 0.12	0.20 (0.10)					2e-30* (HFD+Fru)
	HFD+Fru	0.15 ± 0.10	0.10 (0.08)					

* Abbreviations: CTFC, corrected total cell fluorescence; SD, standard deviation; IQR, interquartile range; SW, Shapiro-Wilk test for normality of distribution; B, Bartlett's test for homogeneity of variances; F, ANOVA test; KW, Kruskal-Wallis rank sum test. *The test was statistically significant (p-value < 0.05).

Table S5. Statistical comparisons of Glands and Villi SSR values between normal diet (ND), high-fat diet (HFD), and HFD+Fru groups in adult mice.

SSR	Group	Mean ± SD	Median (IQR)	F (p-value)	SW (p-value)	B (p-value)	p-values from post hoc tests
Glands	ND	0.92 ± 0.05	0.93 (0.06)	0.594 (0.554)	0.973 (0.663)	5.244 (0.073)	-
	HFD	0.91 ± 0.07	0.92 (0.10)				
	HFD+Fru	0.93 ± 0.06	0.93 (0.08)				
Villi	ND	0.93 ± 0.04	0.92 (0.05)	11.76 (3e-05*)	0.974 (0.080)	2.915 (0.233)	0.0007* (HFD); 0.00006* (HFD+Fru)
	HFD	0.89 ± 0.05	0.88 (0.05)				0.701 (HFD+Fru)
	HFD+Fru	0.88 ± 0.03	0.88 (0.05)				

* Abbreviations: SSR, secretion rate; SD, standard deviation; IQR, interquartile range; SW, Shapiro-Wilk test for normality of distribution; B, Bartlett's test for homogeneity of variances; F, ANOVA test; Tukey's post hoc test for multiple comparisons of means. *The test was statistically significant (p-value < 0.05).

Table S6. Statistical comparisons of Glands and Villi PAS values between normal diet (ND), high-fat diet (HFD), and HFD+Fr groups in adult mice.

PAS	Group	Mean ± SD	Median (IQR)	KW (p-value)	SW (p-value)	B (p-value)	p-values from post hoc tests
Glands	ND	1.85 ± 0.17	1.93 (0.32)	1.811 (0.404)	0.906 (7.1e-05*)	7.829 (0.020*)	-
	HFD	1.82 ± 0.13	1.84 (0.24)				
	HFD+Fru	1.85 ± 0.09	1.88 (0.10)				
Villi	ND	2.04 ± 0.07	2.06 (0.12)	32.930 (7e-08*)	0.960 (0.012*)	2.330 (0.312)	0.520 (HFD); 6e-08* (HFD+Fru)
	HFD	2.01 ± 0.06	1.98 (0.09)				8e-04* (HFD+Fru)
	HFD+Fru	1.92 ± 0.06	1.93 (0.06)				

* Abbreviations: PAS, periodic acid-Schiff; SD, standard deviation; IQR, interquartile range; SW, Shapiro-Wilk test for normality of distribution; B, Bartlett's test for homogeneity of variances; KW, Kruskal-Wallis rank sum test. *The test was statistically significant (p-value < 0.05).
